# Adsorption and Electropolymerization of *p*-Aminophenol Reduces Reproducibility of Electrochemical Immunoassays

**DOI:** 10.3390/molecules27186046

**Published:** 2022-09-16

**Authors:** Grace Buckey, Olivia E. Owens, Ainslee W. Gabriel, Claudia M. Downing, Margaret C. Calhoun, David E. Cliffel

**Affiliations:** 1 Department of Chemistry, Vanderbilt University, 7330 Stevenson Center, VU Station B 351822, Nashville, TN 37235, USA; 2 United States Naval Academy, 121 Blake Rd, Annapolis, MD 21402, USA

**Keywords:** *p*-aminophenol, electrochemistry, electropolymerization, adsorption, screen-printed electrode, square wave voltammetry

## Abstract

This paper investigates the electrochemical behavior of *p*-aminophenol (PAP) on commercially available carbon screen-printed electrodes (CSPEs) and gold screen-printed electrodes (GSPEs) at neutral and basic pHs for the development of inexpensive immunoassays. The electrochemical oxidative signal from PAP results from its adsorption to the electrode. The formation of self-assembled monolayers on gold electrodes prevented PAP adsorption but also reduced its oxidative current, confirming that adsorption increases signal production. On bare electrodes, PAP adsorption results in oxidative current variability depending on the electroactive surface area of the screen-printed electrode. This variability could not be remedied by cleaning and reusing the same GSPE. Decreasing the PAP concentration to 3.8 μM greatly improved the consistency of the measurements, suggesting that the adsorption of PAP is concentration-dependent. Multiple PAP oxidations on the same electrode caused polymerization, limiting PAP in continuous monitoring applications. Infrared and Raman spectroscopy allow the distinction between adsorbed PAP and electropolymerized PAP on the surface of a gold wafer. The results from this study suggest that the use of PAP production in immunoassays with SPEs must be fine-tuned, and electrodes must be cleaned or disposed of between measurements.

## 1. Introduction

Electrochemical immunoassays are a class of biosensors that combine high specificity, low detection limits, and low cost [1]; this is achieved by capitalizing on strong antibody–antigen interactions and the inexpensive nature of electrochemical instrumentation. These features make electrochemical immunoassays well-suited for point-of-care device development, making human health monitoring more accessible. Electrochemical assays couple analyte binding with electrochemical signal amplification by incorporating an enzyme/substrate pair.

*p*-aminophenyl phosphate (PAPP) is a commonly used enzymatic substrate for the enzyme alkaline phosphatase, which hydrolyzes PAPP into *p*-aminophenol (PAP) [1,2,3,4]. PAP exhibits promising electrochemical properties, such as a low oxidation potential and electrochemical reversibility, making it an appealing candidate for assay design [5,6]. Furthermore, while still electrochemically active, PAPP demonstrates an oxidation potential above that of PAP [7], making it possible to develop electrochemical immunoassays using the PAPP/PAP system.

**Scheme 1:**
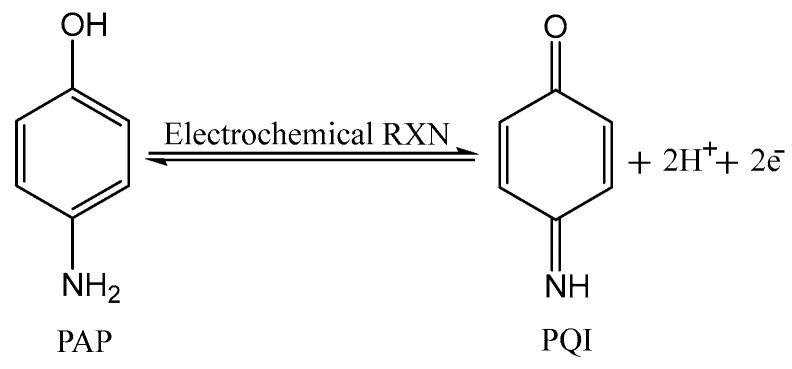
Reaction mechanism of PAP oxidation.

Groups that use the PAPP/PAP system have reported that PAP undergoes a reversible two-proton–two-electron reaction at the working electrode to produce p-quinone imine (PQI) (Scheme 1) [7]. However, a literature review revealed that the oxidation of PAP is not as straightforward as this scheme suggests. Electrochemical studies of the oxidation of PAP have been conducted in organic media [8,9,10], at various pHs in aqueous media [7,11,12,13,14], and on gold [8,10], platinum [9,11], mercury [15], glassy carbon [7,16], carbon paste [2,3,14], and graphite [12] electrodes. These studies reported slightly different reaction mechanisms and products, emphasizing the impact of reaction conditions [12]. Some accounts reported a diffusion-controlled [3] oxidation process, while others reported film formation [13]. Others reported that PAP oxidation resulted in electropolymerization on the surface of the electrode [8,9,12]. These competing reactions complicate the use of PAP in assays and could affect the reproducibility of assay measurements that rely on the production of PQI for the readout. As a result, it is necessary to characterize PAP oxidation under the reaction conditions used in assays.

Due to the enzymatic activity of alkaline phosphatase being approximately three times greater at pH 9 than pH 7, many immunoassays use pH 9 for the PAPP/PAP system [1,2,3,4]. Different pH causes variation in the protonation of the hydroxyl and amine groups of PAP, producing different polymeric films [12,17]. Some analyses that focused on acidic pH reported the formation of a polymer [12,13], while others did not [10,11]. Analyses at basic pH reported film formation [13], some due to polymerization [12], while studies that focused on PAP’s oxidative behavior at neutral pH [14,18,19] did not report polymer formation. However, one study at neutral pH reported that oxidation products caused electrode fouling [18]. 

The purpose of this study was to investigate PAP’s behavior at neutral and basic pHs on commercially available carbon screen-printed electrodes (CSPE) and gold screen-printed electrodes (GSPEs) (Pine Research Instrumentation, Inc.) to close the gap between assay development using PAP and electrochemical characterization of PAP. CSPEs and GSPEs are inexpensive, can measure small volume samples, and are ideal for developing point-of-care devices. CSPEs are particularly affordable, allowing for the development of disposable immunoassays; this is of great interest in biosensor development as biological samples can often foul the electrode surface. Rather than performing rigorous cleaning procedures to avoid fouling issues, these electrodes can simply be disposed of and replaced. While GSPEs do not have the advantage of being disposable, they are designed to be reusable and withstand rigorous cleaning procedures. These characteristics could be beneficial in preventing interference from electrode fouling, allowing the same electrode to be used for multiple measurements.

Our results show that the adsorption and polymerization of PAP complicate its use in assay development. Utilizing a self-assembled monolayer to prevent PAP adsorption reduced the electrochemical oxidation current, confirming that adsorption is required for signal output at the concentrations used. Relying on PAP’s adsorption to obtain signal resulted in widely variable signals across both CSPEs and GSPEs due to inconsistent adsorption to the different surface morphologies of electrodes. The GSPEs showed considerable variability in peak currents even when using the same electrode and cleaning between scans. Concentrations of 25 µM PAP were initially investigated as higher concentrations would allow for an extended linear range of detection. However, the adsorption issues of PAP can be mitigated using lower concentrations of PAP. Limiting PAP production in immunoassays is necessary to ensure the reproducibility of PAP oxidation and assay measurements. Multiple, subsequent measurements on one electrode resulted in the polymerization of PAP on both CSPEs and GSPEs, preventing this system from being useful for continuous monitoring. 

Polarization modulation-infrared reflection absorption spectroscopy (PM-IRRAS) and Raman spectroscopy were used to characterize and distinguish between adsorbed and polymerized PAP. Previous groups have characterized the PAP monomer, PAP polymer, and PAP films using infrared spectroscopy [8,9,11,13], consistent with our PM-IRRAS of adsorbed and polymerized PAP. Using Raman spectroscopy, other groups have characterized the PAP monomer [20] and a PAP radical cation [21]. However, to the best of our knowledge, we are the first to report a Raman spectrum of the PAP polymer. The PM-IRRAS and Raman spectra confirmed the presence of adsorbed PAP and PAP polymer on the gold’s surface and provided a distinction between the two species.

## 2. Results

### 2.1. Electrochemical Characterization of p-Aminophenyl Phosphate

**Figure 1:**
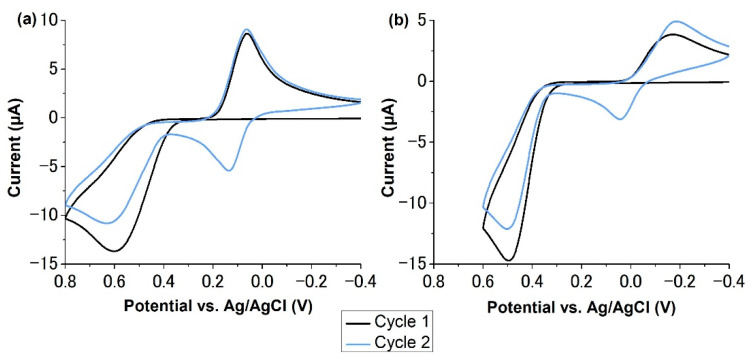
Cyclic voltammograms of 5 mM PAPP in Tris buffer at (**a**) pH 7 and (**b**) pH 9. An extra oxidation peak emerged in the second cycle of both, indicating the presence of PAP.

The electrochemical behavior of 5 mM PAPP, a commonly used concentration in assay development, was examined using cyclic voltammetry on bare CSPEs at pHs 7 and 9 (Figure 1). At pH 7, PAPP was oxidized at 0.573 V and reduced at 0.063 V vs. Ag/AgCl in the first cycle (Figure 1a). In the first cycle at pH 9, PAPP was oxidized at 0.498 V and reduced at −0.172 V vs. Ag/AgCl (Figure 1b). Scanning at these high oxidative potentials non-enzymatically generated PAP to produce an additional oxidation peak in the second cycle; this occurs via an ECE mechanism, where the “E” represents an electron transfer at the electrode surface, and “C” represents a chemical reaction [22]. The initial PAPP oxidation results in the chemical conversion of PAPP to PAP, which allows for a second electron transfer from the subsequent PAP oxidation within the same potential window. These additional oxidation peaks can be seen at 0.136 V at pH 7 and 0.043 V at pH 9. Thus, if scanning for multiple cycles, the oxidation switching potential is important when using the PAPP/PAP system. Allowing the potential to reach 0.3 V at pHs 7 and 9 yielded small amounts of non-enzymatic electrochemical produced PAP from PAPP at the foot of the PAPP oxidation wave (Figure S1 Supplementary materials). This current is negligible compared to the oxidative current from enzymatically produced PAP; however, it is still important to consider to avoid interference from the unintended production of PAP from repetitive scanning.

### 2.2. The Effects of Sonication on p-Aminophenyl Phosphate

**Figure 2:**
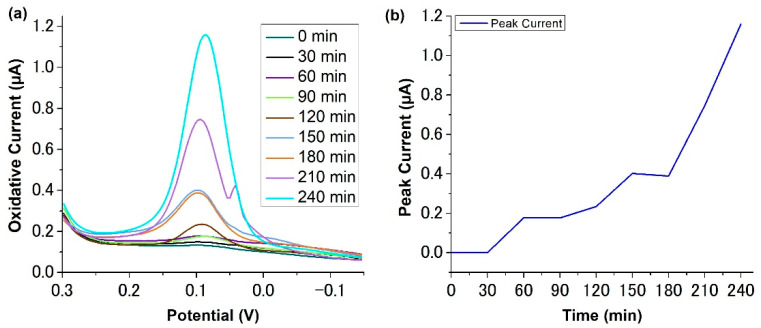
(**a**) Square wave voltammograms of 5 mM PAPP (pH 7) at increasing sonication times on a GSPE and (**b**) peak current vs. sonication time. An increase in peak current occurs with increasing sonication time.

Sonication is a common technique used to ensure the complete dissolution of an analyte. Studies were conducted to identify the effects of sonication on PAPP’s electrochemical behavior. Square wave voltammograms (SWVs) were taken of 5 mM PAPP solutions that were sonicated for times ranging from 10 min to 4 h (Figure 2). These studies were completed using GSPEs to ensure that proper cleaning of the working electrode was achieved between scans, preventing interference from electrode fouling. It was determined that sonication for 30 min or less did not affect PAPP’s electrochemical behavior in this window. However, sonication times of 1 h or longer yielded PAP oxidation peaks, suggesting the non-enzymatic production of PAP from PAPP sonication. Furthermore, increasing sonication time resulted in a larger peak size, suggesting that longer sonication times generated higher concentrations of PAP. Therefore, PAPP should not be sonicated for longer than 30 min to prevent non-enzymatic conversion and interference in assay measurements.

It was also determined that solutions of PAPP non-enzymatically produce PAP over time; this was evident either electrochemically or by yellowing (photo-oxidation) of the solutions. Phenols are known to photo-oxidize, resulting in the solution’s color change [23]. Therefore, the yellowing of PAPP solutions results from the photo-oxidation of PAP, demonstrating the presence of PAP in the solution. Additionally, the photo-oxidation of PAP occurs faster at pH 9 than at pH 7. Furthermore, keeping PAPP solutions refrigerated and in dark conditions when not in use slowed the degradation process. However, degradation was still evident if PAPP solutions were kept longer than approximately one week. As a result, solutions of PAPP should be made fresh each week.

### 2.3. Adsorption of p-Aminophenol

**Figure 3:**
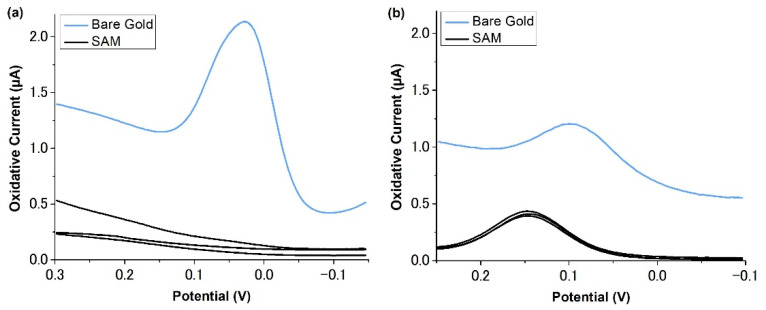
SWVs of (**a**) 25 uM PAP on a bare gold disk electrode and three different disk electrodes modified with a 3-mercaptopropanol SAM and (**b**) 25 uM FcMeOH on a bare gold disk electrode and three different disk electrodes modified with a 3-mercaptopropanol SAM.

A 25 µM sample of PAP in Tris buffer at pH 7 was investigated via SWV on a bare gold disk electrode and gold disk electrodes coated with a thin self-assembled monolayer (SAM). The bare electrode yielded a prominent PAP oxidation peak, while three separate 3-mercaptopropanol-modified electrodes yielded no peaks (Figure 3a). Additionally, 25 µM ferrocenemethanol (FcMeOH) in Tris buffer at pH 7 was investigated on a bare gold disk electrode and on three separate 3-mercaptopropanol-modified disk electrodes to confirm that the SAM does not prohibit electron transfer to the electrode surface (Figure 3b).

FcMeOH gave replicable peaks, confirming that the SAM allowed sufficient electron transfer from the species in the solution. Furthermore, the peak current for FcMeOH remained consistent between SAM-modified and bare gold electrodes, although the baselines were shifted; this confirms that the SAM blocks signal arising from adsorption. The absence of PAP peaks on SAM-coated electrodes further shows that the SAM prevented PAP adsorption to the electrode surface; however, it also suggests that PAP detection at this concentration depends on its adsorption.

PAP should give peak currents twice that of FcMeOH as it undergoes two-electron oxidation compared to FcMeOH’s one-electron oxidation. However, in our data, PAP on bare gold has a peak current 3.8 times larger than FcMeOH on bare gold (Figure 3). This discrepancy is likely due to the adsorption of PAP on the electrode surface augmenting the signal.

**Figure 4:**
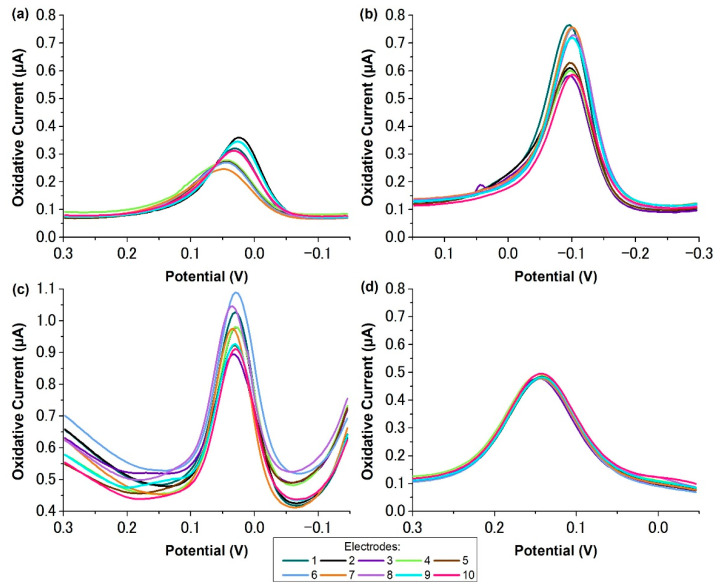
SWVs of (**a**) 25 μM PAP at pH 7 on ten different CSPEs, (**b**) 25 μM PAP at pH 9 on ten different CSPEs, (**c**) 25 μM PAP at pH 7 on ten different GSPEs, and (**d**) 25 μM FcMeOH on ten different CSPEs.

Moreover, 25 µM PAP in Tris buffer was analyzed on ten CSPEs at pHs 7 and 9 and on ten GSPEs at pH 7. The signal varied up to 41% for the CSPE at pH 7 (Figure 4a), 39% for the CSPE at pH 9 (Figure 4b), and 39% for the GSPE at pH 7 (Figure 4c). The variability in the signal of PAP suggests that the SPEs have different microscopic surface areas. The variance in signal between CSPEs and GSPEs was similar, but the baseline of the GSPEs was less consistent. Adsorption issues were not limited to SPEs and were also observed on gold disk electrodes. FcMeOH was used as the control because it remains unaffected by the differing electroactive surface areas between electrodes due to its outer sphere electron transfer mechanism. As expected, the FcMeOH signal only varied 7.5% between the ten scans (Figure 4d).

**Figure 5:**
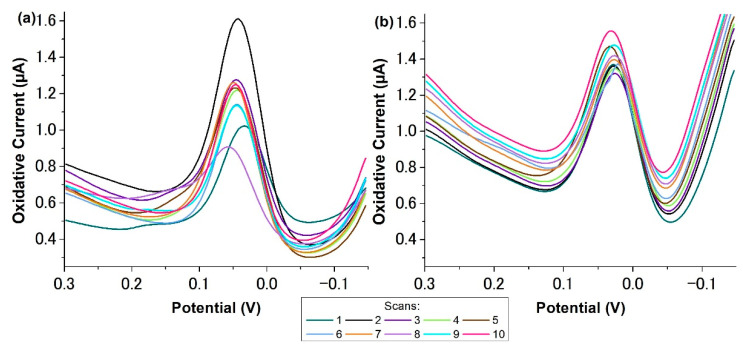
Ten SWVs with cleaning between each scan of (**a**) 25 μM PAP on a single GSPE and (**b**) 3.8 μM PAP on a single GSPE. At 25 µM, a complete coating of PAP to the GSPE resulted in a large percentage difference of 88% between the smallest and largest peaks. Decreasing the PAP concentration to 3.8 µM improved the reproducibility to a 19% difference between the smallest and largest peaks.

Analyses of 25 μM PAP were performed via SWV ten separate times on a single GSPE with cleaning between scans (Figure 5a). An 88% difference was observed between the smallest and largest peaks. Although the cleaning procedure prevented electrode fouling, it did not resolve the issue of irreproducibility across multiple scans. A complete PAP coating during oxidation would result in different peak sizes depending upon the different electroactive surface areas. To test if the contribution of electroactive surface area to peak irreproducibility could be minimized with less PAP adsorption, the PAP concentration was decreased to 3.8 μM. Ten SWVs were retaken on a single GSPE with cleaning in between (Figure 5b). PAP showed improved reproducibility at this lower concentration, with a 19% difference between the smallest and largest peaks, likely due to an incomplete electrode coating. These results suggest that the varied adsorption of PAP is concentration-dependent, and variability resulting from differing electrode morphology may be improved by restricting PAP to low concentrations.

### 2.4. Electropolymerization of p-Aminophenol

**Figure 6:**
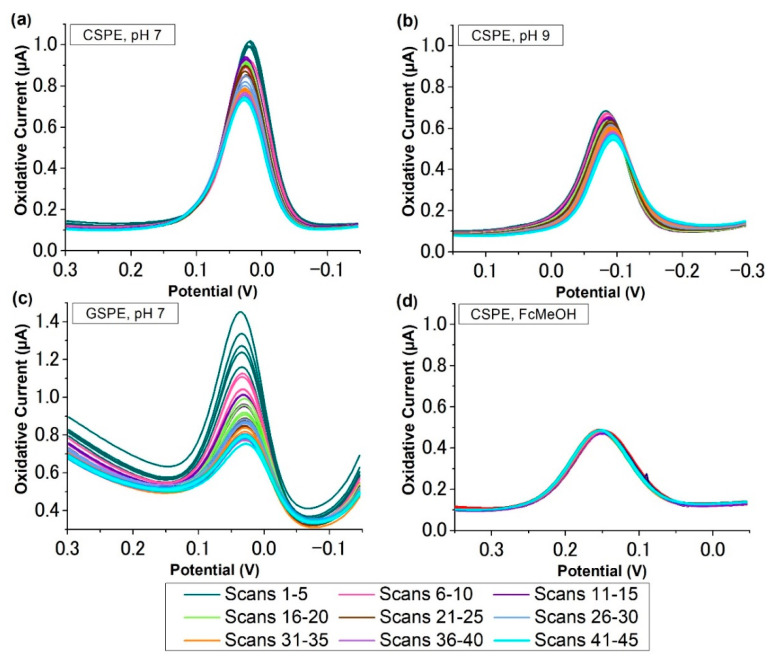
Forty-five SWVs of (**a**) 25 μM PAP at pH 7 on a CSPE, (**b**) 25 μM PAP at pH 9 on a CSPE, (**c**) 25 μM PAP at pH 7 on a GSPE, and (**d**) 25 μM FcMeOH on a CSPE.

To determine the suitability of CSPEs and GSPEs for multiple scans of PAP at both neutral and basic pHs, 45 SWV scans of PAP on new CSPEs and a GSPE were compared to 45 SWV scans of FcMeOH on one CSPE. The PAP scans on CSPEs and GSPEs at neutral and basic pHs showed a decrease in peak current with an increasing number of scans, suggesting that an insulating polymer was formed. The SWV peaks decreased up to 34% for the CSPE at pH 7 (Figure 6a), 28% for the CSPE at pH 9 (Figure 6b), and 92% for the GSPE at pH 7 (Figure 6c). The CSPEs exhibited much less signal suppression than the GSPEs, suggesting that gold promotes the formation of the PAP polymer. However, despite the signal suppression being less severe with the CSPEs, the large decrease in peak size prevents either electrode from being suitable for repeated measurements at this concentration. Polymerization was not limited to SPEs and also occurred on gold disk electrodes. PAP polymerization was also investigated using non-reversal electrochemical techniques to determine if the reverse pulses used in SWV promoted PAP polymerization. Unfortunately, polymerization could not be avoided using non-reversal techniques. FcMeOH showed no significant change in peak current, giving a variance of 3.0% between the smallest and largest values (Figure 6d); this was expected because FcMeOH undergoes an outer sphere electron transfer and does not electropolymerize.

### 2.5. Spectral Analysis of Adsorbed PAP and PAP Polymer 

**Figure 7:**
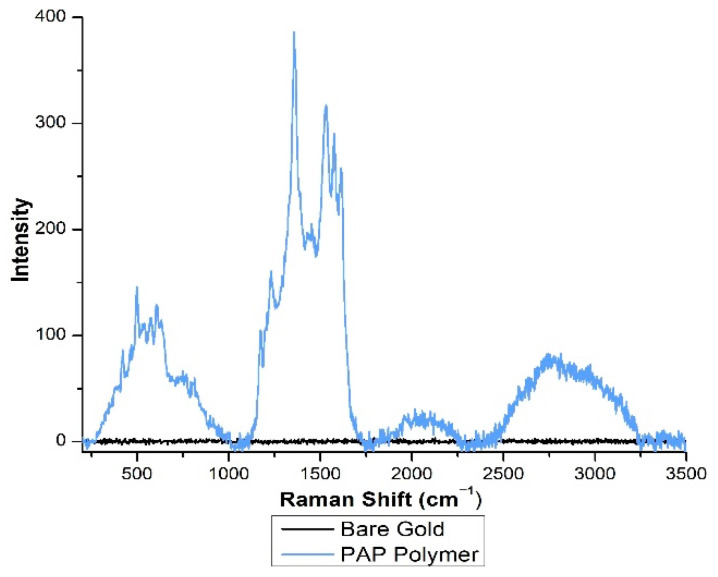
Raman spectra of PAP polymerized on a gold wafer (blue) and a bare gold wafer (black).

To confirm the presence of adsorbed PAP and polymeric PAP on the surface of the electrode, polarization modulation-infrared reflection absorption spectroscopy (PM-IRRAS) was conducted on polymerized PAP and adsorbed PAP with bare gold as a control. Additionally, Raman spectroscopy was conducted on polymerized PAP with bare gold as a control. Infrared spectroscopy of the PAP monomer and polymer has previously been reported [8,9,11,13], as has Raman spectroscopy of the PAP monomer [20] and a PAP radical cation [21]. However, to the best of our knowledge, this is the first reported Raman spectrum of the PAP polymer. PM-IRRAS absorption bands of the adsorbed PAP were consistent with previously reported PAP monomer absorption bands characteristic of aromatic compounds [9] and are shown in the Supplementary Materials (Figures S2 and S3, and Table S1). The polymer demonstrated PM-IRRAS bands consistent with aromatic vibrations, specific linkages, and functional groups defined in previously reported PAP polymer characterization studies [8,11,13]. The differences in PM-IRRAS spectra between adsorbed PAP and PAP polymer confirmed a clear distinction between the two species. Raman absorption bands of the PAP polymer (Figure 7) were compared to previously published spectra of the PAP monomer and PAP radical cation (Table S2). The PAP polymer shared aromatic bands at 1613 cm^−1^, 1453 cm^−1^, 1358 cm^−1^, 1232 cm^−1^, and 1174 cm^−1^ with PAP and PAP cation species [20,21], suggesting that the polymer retains its aromaticity. Previously reported IR spectra of a PAP polymer show that it links via nitrogen and oxygen [9]. We suspect that Raman bands at 604 cm^−1^, 575 cm^−1^, 498 cm^−1^, and 421 cm^−1^ result from those nitrogen and oxygen linkages. 

## 3. Discussion

The data from this study reveal two mechanisms of non-enzymatic PAPP conversion. First, when measuring a sample mixture of PAPP/PAP, the potential window must be carefully selected as the direct electrochemical oxidation of PAPP results in the production of PAP. Second, solutions of PAPP must be made fresh, kept refrigerated, and stored in dark conditions as it degrades to PAP over approximately 1 week. Additionally, PAPP solutions cannot be sonicated as sonication produces PAP. These non-enzymatic conversions of PAPP would lead to residual PAP detection that is not a result of the electrochemical assay being performed. These conclusions should be carefully considered when using PAPP and PAP.

The results from this study also reveal two main issues with the electrochemical oxidation of PAP: adsorption and polymerization. Achieving a detectable amount of electrochemical oxidation from 25 µM PAP relies on its adsorption to the electrode surface. A 3-mercaptopropanol SAM formed on gold electrodes prevented PAP adsorption, but the SAM-modified electrodes could not measure PAP oxidation; this suggests that forcing the detection of non-adsorbed PAP at this concentration does not allow for a measurable signal. Moreover, the adsorption of PAP across different bare carbon or gold electrodes causes localized concentration differences due to the variable electrode morphology, leading to inconsistent and misleading peak sizes. A potential solution for the variable adsorption across different GSPEs was to clean a single GSPE between each measurement. However, cleaning the electrode between each scan at a PAP concentration of 25 μM still demonstrated adsorption inconsistencies. When challenging the GSPE with lower concentrations of PAP, the variance from adsorption was minimized. We theorize that this depreciation is due to an incomplete coating of PAP on the surface of the electrode, mitigating the effects of the differing electroactive surface areas caused by cleaning. The effect of concentration-dependent fouling is not unprecedented and has been reported by Tang and coworkers in the electrochemical oxidation of phenol [5]. Therefore, the concentration of PAP must be carefully tuned if CSPEs and GSPEs are to be used in the development of immunoassays that use PAP for electrochemical readout. 

Multiple scans of PAP on the same electrode result in its polymerization. This fouling prevents one electrode from being used for multiple, subsequent scans, and PAP use as a redox reporter in a continuous monitoring sensor. PM-IRRAS and Raman spectroscopy confirmed the presence of both adsorbed PAP and polymerized PAP on the gold surface. The PM-IRRAS spectrum in this study is consistent with previously published literature, while the Raman data present a new perspective on the polymerized product. The presence of these two species on the surface of a gold wafer further confirms the dual problem of polymerization and adsorption that PAP exhibits.

## 4. Materials and Methods

### 4.1. Materials

All reagents were used as received without additional purification and were obtained from Sigma Aldrich (St. Louis, MO, USA). Tris buffer consisted of 10 mM Trizma^®^ base, 100 mM NaCl, and 1 mM MgCl_2_. The final pH of the buffer was adjusted to either 7 or 9 using HCl. All solutions of *p*-aminophenol (≥98%), *p*-aminophenyl phosphate (monosodium salt; ≥98%), and ferrocenemethanol (≥97%) were made directly in Tris buffer and diluted as necessary. 

### 4.2. Instrumentation

Electrochemical measurements were performed with CHI 1440 4-Channel Potentiometer/Potentiostat (CH Instruments, Inc., Austin, TX, USA). Carbon screen-printed electrodes from Pine Research Instrumentation, Inc. with a 2 mm diameter carbon working electrode, a Ag/AgCl reference electrode, and a carbon counter electrode were used for measurements. The experiments were carried out on a bare activated CSPE. Sonication studies were conducted on bare gold screen-printed electrodes from Pine Research Instrumentation, Inc. (Durham, NC, USA) with a 2 mm diameter gold working electrode, a Ag/AgCl reference electrode, and a gold counter electrode. Self-assembled monolayer experiments were conducted with gold disk electrodes from CH Instruments, Inc. (Austin, TX, USA) with a 2 mm diameter gold working electrode, a Ag/AgCl reference electrode, and a gold counter electrode.

PM-IRRAS measurements were taken with a Bruker Tensor 27 FTIR (Bruker Corporation, Billerica, MA, USA) equipped with PMA 50. In addition, Raman spectroscopy measurements were taken with a Thermo Scientific DXR Confocal Raman microscope (Thermo Fisher Scientific, Waltham, MA, USA). These measurements were taken on gold-coated silicon wafers with titanium adhesion layers of approximately 1 cm × 1 cm (Sigma 643262).

### 4.3. Analytical Procedures

CSPEs were activated in 10 mL of 0.05 M H_2_SO_4_ by cyclic voltammetry, scanning from −1.5 to 1.5 V at a scan rate of 100 mV/s for 5 cycles. GSPEs were cleaned in 10 mL of 0.5 M H_2_SO_4_ by cyclic voltammetry, scanning from −0.375 to 1.8 V at a scan rate of 500 mV/s for 10 cycles. 

Gold wafers were electrochemically cleaned by cycling in 0.5 M H_2_SO_4_ for 10 cycles. Polymerization was conducted in 10 mL of 5 mM PAP on a gold wafer using chronoamperometry for 13 min with a potential of 0.3 V. Adsorption was also conducted in 10 mL of 5 mM PAP by placing a gold wafer in 10 mL of 5 mM PAP for 13 min. 

All experiments on SPEs were conducted by drop casting 100 uL of the sample. All experiments performed with disk electrodes were conducted in 5 mL of the sample. The self-assembled monolayer-modified gold disks were made by submerging them in 30 mM 3-mercaptopropanol for 20 h.

## 5. Conclusions

The oxidative signal of PAP at neutral and basic pHs depends on its adsorption to the electrode. Coating gold electrodes in a self-assembled monolayer resulted in no signal, confirming that PAP oxidation at concentrations of 25 µM and below depends on adsorption. When attempting to use a bare CSPE or GSPE for each PAP measurement or cleaning GSPEs in between each measurement, the various microscopic surface area of the electrodes resulted in widely variable peak currents due to inconsistent PAP adsorption. The variable adsorption of PAP is concentration-dependent and can be minimized by decreasing the PAP concentration. PAP cannot be used for a continuous monitoring sensor as multiple scans of PAP on the same electrode result in polymerization. Overall, this study suggests that when developing immunoassays using CSPEs and GSPEs with the PAPP/PAP system, careful consideration must be taken to control the concentration of PAP produced and minimize the effects of PAP adsorption. Additionally, electrodes must be used in a disposable manner or cleaned between measurements to prevent polymerization of PAP on the electrode surface. 

## Data Availability

Not applicable.

## Supplementary Materials

The following supporting information can be downloaded at: https://www.mdpi.com/article/10.3390/molecules27186046/s1, Figure S1: Cyclic voltammogram switching potential for PAPP; Figure S2: Overlayed PM-IRRAS spectra with absolute intensities of PAP polymerized on a gold wafer, adsorbed PAP on a gold wafer, and a bare gold wafer; Figure S3: Normalized PM-IRRAS spectra of PAP polymer and adsorbed PAP; Table S1: IR absorption (cm^−1^) of adsorbed PAP and PAP polymerized by chronoamperometry compared to previously reported PAP monomer and polymer; Table S2: Summary of the Raman absorption bands of the adsorbed PAP and PAP polymer citing corresponding supporting literature.

## Sample Availability

Samples of the compounds are not available from the authors.
