# Supplementary material for: Adsorption and Electropolymerization of p-Aminophenol Reduces Reproducibility of Electrochemical Immunoassays
Source: Molecules. 2022 Sep 16;27(18):6046. doi: 10.3390/molecules27186046 (PMC9501838; doi:10.3390/molecules27186046)
Supplement: Supplementary file 1 [file molecules-27-06046-s001.zip › molecules-1907168-supplementary.pdf]

Supplementary Materials

Adsorption and Electropolymerization of *p*-Aminophenol Reduces Reproducibility of Electrochemical Immunoassays

Grace Buckey [1, †], Olivia E. Owens [1, †], Ainslee Gabriel [2], Claudia Downing [2], Margaret C. Calhoun [1], David E. Cliffel [1*]

1. Department of Chemistry, Vanderbilt University, 7330 Stevenson Center, VU Station B 351822, Nashville, Tennessee 37235-1822, United States; grace.e.buckey@vanderbilt.edu (G.B); olivia.eldridge@vanderbilt.edu (O.E.O)
2. United States Naval Academy. 121 Blake Rd, Annapolis, MD 21402, United States

* Correspondence: d.cliffel@vanderbilt.edu (D.E.C)

† These authors contributed equally to this work. Order of authors was decided alphabetically.

Cyclic Voltammogram Switching Potential for PAPP

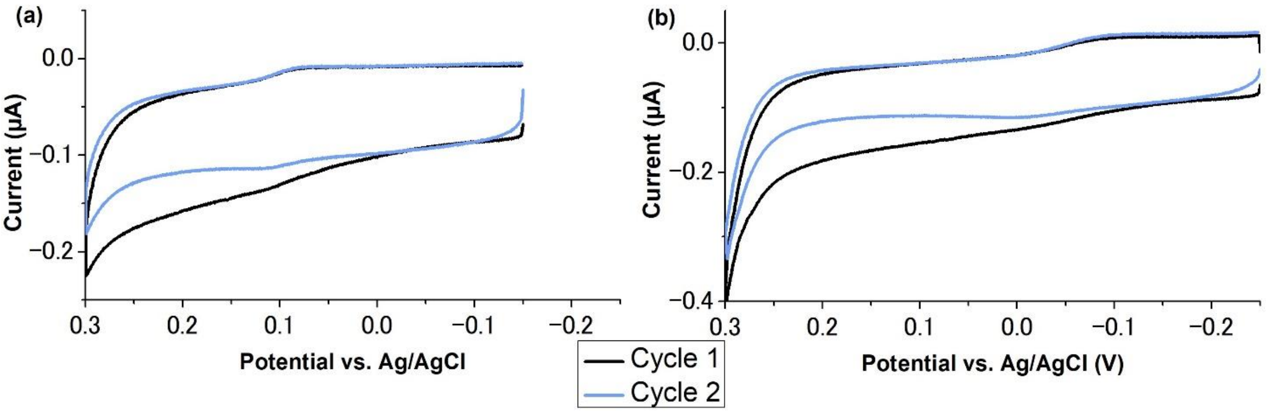

Figure S1. Cyclic voltammograms of 5mM PAPP in Tris buffer with a potential window limited to 0.3 V at a) pH 7 and b) pH 9. These results show the importance of determining the appropriate switching potential to minimize electrochemical production of PAP from repetitive scanning.

PM-IRRAS Analysis of PAP Polymer, Adsorbed PAP, and Bare Gold Wafer

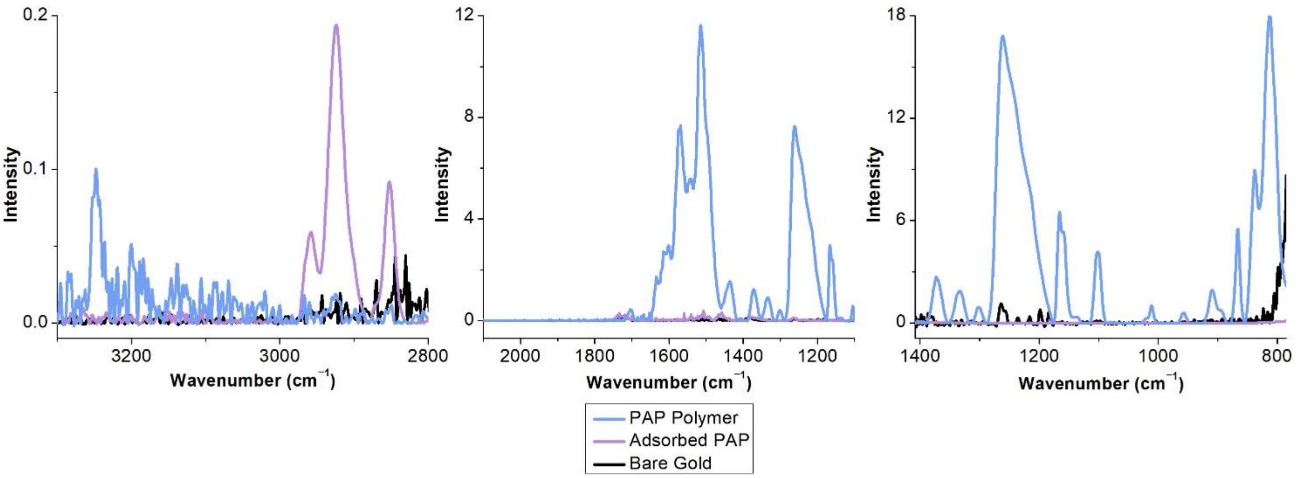

Figure S2. PM-IRRAS spectra with absolute intensities of PAP polymerized on a gold wafer (blue), adsorbed PAP on a gold wafer (purple), and a bare gold wafer (black). The spectra are overlaid to emphasize the increased intensity of the polymer compared to adsorbed PAP.

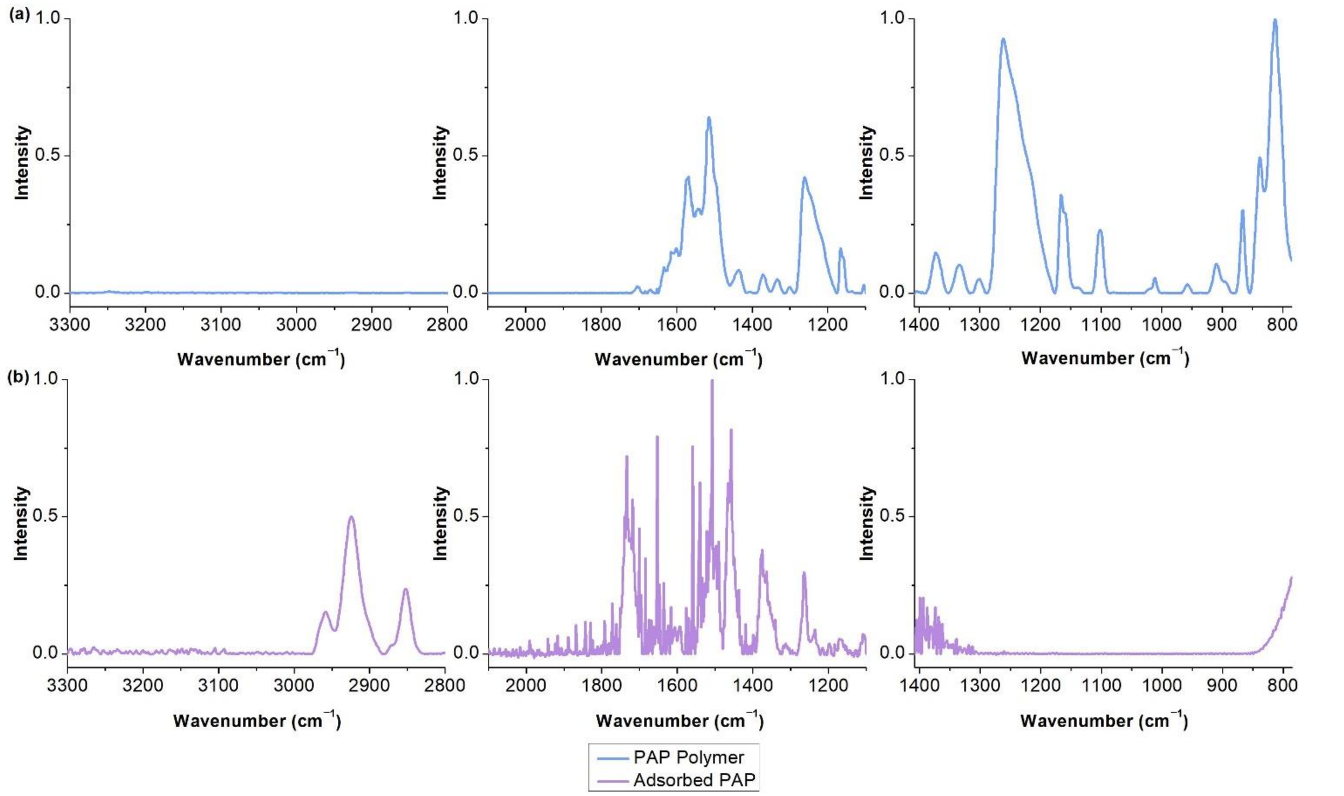

Figure S3. PM-IRRAS spectra of a) PAP polymer and b) adsorbed PAP, both normalized to an intensity of one by dividing by the raw value of the most intense peak

Table S1. Absorption bands (cm-1) of adsorbed PAP and PAP polymerized by chronoamperometry compared to previously reported PAP monomer and polymer.

Vibration/Functional Group	Adsorbed PAP	Previously Reported in
C=C Stretch	2923	[9]
C-H Stretch	2852, 2958	[9]
**Vibration/Functional Group**	**PAP Polymer**	**Previously Reported in**
Phenyl Ring Stretch	1538, 1543, 1370	[13]
C=C Stretch	1600, 1615	[8]
Phenyl Ring Stretch	1600, 1615	[13]
C=C Phenyl Ring Stretch	1514	[7], [8], [13]
C=C Vibration	1612	[8], [9]
Substituted imine	1612, 1634	[9]
O-H deformation or C-O stretching of phenol	1261	[7], [8]
Imine and ether linkages	1159, 1100, 1261	[9]
C-H in plane deformation	1011, 1165	[13]

Raman Analysis Polymerized PAP and Bare Gold Wafer

Table S2. Absorption bands (cm^{-1}) of PAP polymerized by chronoamperometry compared to previously reported PAP species.

Vibration/Functional Group	PAP Polymer	Consistent with	Reported in
C-C Ring Stretch	1232, 1613	PAP	[20]
CH Bend	1174	PAP Cation	[21]
CC Stretch	1358, 1453	PAP Cation	[21]
OH Bend	1531	PAP Cation	[21]
Aromatic Ring Stretch	1575	Unique to polymer	This work
Polymer Linkage	421	Unique to polymer	This work
Polymer Linkage	498	Unique to polymer	This work
Polymer Linkage	575	Unique to polymer	This work
Polymer Linkage	604	Unique to polymer	This work
